# Issue Leadership: Establishing a Domain for a Food Systems Leadership Model

**DOI:** 10.3390/foods12132598

**Published:** 2023-07-04

**Authors:** Kevan W. Lamm

**Affiliations:** Department of Agricultural Leadership, Education & Communication, University of Georgia, Athens, GA 30606, USA; kl@uga.edu

**Keywords:** agriculture, food systems, leadership development, policy, community engagement, interpersonal skills, organizational skills

## Abstract

A sustainable food system is a fundamental requirement for the ongoing functioning and growth of society. However, despite the critical importance of the food system from both economic and social perspectives, there are several political, environmental, and human capital issues which represent barriers to sustainable production. For example, in the United States, the need for more production capacity to feed a growing population is juxtaposed with a shrinking and aging food system workforce. The nexus of such fundamentally opposed issues represents a situation in which technical solutions may be insufficient. Using a three-round Delphi process with an expert panel, a total of 106 unique leadership competencies or behaviors were identified. The resulting behaviors and competencies were then thematically analyzed using the constant comparative method. The proposed food systems leadership model, named Issue Leadership, includes 39 subthemes and 7 primary themes. The primary themes include action; change; communication; critical thinking, strategic planning and visioning; interpersonal traits and characteristics; leadership skills; and leadership processes. This study establishes the unique context that agriculture and food systems represent and the necessity for leadership models that are competency- and practice-based.

## 1. Introduction

The importance and necessity of a robust food system cannot be understated relative to society’s functioning and growth [1]. In modern society, the economic impact of agriculture and natural resources has been quantified. The global agricultural market was estimated to be over USD 13.3 trillion in 2023, a 9.4% increase over 2022 [2]. In 2021, agricultural production had a total economic impact of USD 1.26 trillion in the US and employed over 21.1 million individuals in direct or indirect roles [3]. However, the increased demand for agricultural output is juxtaposed with a number of critical issues the agriculture and natural resources industry has been facing (e.g., [4,5,6,7,8]), “transformative change in agriculture and food systems are required worldwide” [9] (p. vii). As Hofman-Bergholm [10], states, “Transformation, education and innovation in different disciplines will be a necessity for a secure and sustainable food system in the future” (p. 11).

### 1.1. Occupations and the Uniqueness of Food Systems Work

Over sixty years ago, Weiss and Kahn [11] stated, “It seems reasonable to expect difference in definition of work as we go from occupation to occupation, since occupations differ so sharply in the kinds of responsibility they demand” (p. 142). It is from this perspective that the interrelated network of occupations affiliated with food systems is considered. For example, the United States Bureau of Labor Statistics [12] uses a classification system in which “workers are classified into occupational categories based upon the work they perform and their skills, education, training, and credentials” (para. 1). Such occupations are linked to industries that represent sectors of the economy. The United States Census Bureau North American Industry Classification System (NAICS) [13] identifies the Agriculture, Forestry, Fishing and Hunting (Agriculture) sector as “primarily growing crops, raising animals, harvesting timber, and harvesting fish and other animals from a farm, ranch, or their natural habitats” (para. 1).

The NAICS framework [13] is hierarchical, beginning with either Goods-Producing Industries or Service-Providing Industries at the top level. The sectors are then grouped into eleven supersectors: three in Goods Producing and the remaining eight in Service Providing. The supersectors subsume the 20 distinct NAICS sectors. The Goods-Producing area includes the Natural Resources and Mining supersector, which contains the Agriculture sector. As an alternative example, the Service-Providing area includes the Education and Health Services supersector, which contains the Health Care and Social Assistance sector. The distinctness of the occupational responsibilities associated with different industries is therefore well established in practice [11]. Additionally, these findings have been examined theoretically, with the previous literature finding that different occupational responsibilities and the associated industries are germane and should inform research [14,15].

### 1.2. Food Systems Facing Significant Issues

Based on readily observable demographic trends, the global population has been facing a confluence of potentially devastating factors [9,16,17]. While the global population is projected to steadily increase [18], requiring the agricultural community to dramatically increase production by 2050 [19], the number of individuals engaged in the agriculture and natural resource industry in the United States has steadily declined [7,8,20]. Overall, there are fewer members of society engaged in agriculture and natural resources [7]. In parallel, the number of policies and regulations directed at the agriculture and natural resource industry has increased. In the United States, agricultural production may require compliance with a wide range of such regulations, including the “Clean Water Act, the Endangered Species Act, the Federal Insecticide, Fungicide and Rodenticide Act, the Food Safety Modernization Act, immigration and labor regulations, and interpretation of the Federal Land Policy and Management Act” [21] (para. 3), a likely consequence of dense urban community populations, which have the ability to direct policy without understanding the potential consequences (e.g., [22]). These trends are also visible globally. According to the Organisation for Economic Co-operation and Development (OECD) [23], “Most current support policies are not serving the wider needs of food systems”, and “Agricultural support policies have failed to address rapid structural change across food systems and the problems these changes have induced” (p. 2).

### 1.3. Existing Leadership Theory Summary

As Northouse [24] states, “there are almost as many different definitions of leadership as there are people who have tried to define it” (p. 2). Therefore, the intent of the following section is not to replace the alternative literature which already summarizes the topic in much greater depth (see [24,25]). However, despite the scope of leadership and leadership development as a discipline, there is no single comprehensive theory that has been adopted [26]. Previous research has attempted to integrate existing theories into superordinate categories of leadership competency. For example, Fleishman et al. [27] identified information search and structuring, information use in problem solving, managing personnel resources, and managing material resources as core across many leadership theories. Alternatively, Yukl et al. [28] identified three primary leadership areas including task, relations, and change. However, the challenge with all-encompassing models or theories is the dilution of utility specifically. In this regard, the challenge mirrors the bandwidth–fidelity dilemma espoused by Cronbach and Gleser [29]. Specifically, “(1) broad, global constructs ought to predict broad criteria with moderate validity; and (2) narrow, specific constructs ought to predict specific criteria with maximal validity” [30] (p. 611). From this perspective, defining specific contexts should improve precision when aligning constructs and criteria, including within leadership contexts [31].

Within the existing literature, there are analyses linking criteria (context) and construct (leadership theory). For example, Piwowar-Sulej and Iqbal [32] performed a systematic literature review examining sustainable performance (criteria) based on leadership styles (construct). The researchers found several studies that employed contemporary leadership approaches such as transformational leadership, with factors including inspirational motivation, idealized influence, individualized consideration, and intellectual stimulation [33]; servant leadership [34], with factors including putting followers first, emotional healing, conceptualizing, helping followers grow and succeed, empowering followers, behaving ethically, and creating value for the community [35]; and authentic leadership [36], with factors including balanced processing, internalized moral perspective, self-awareness, and relational transparency [37]. However, the researchers found that many of the common leadership theories in the literature are rooted in business management and are therefore not necessarily predictive of sustainable performance. Additionally, the researchers recommended that future research examine leadership styles adapted to specific contexts [32].

### 1.4. Leadership as Theory and Practice in Context

Based on recommendations in the literature for leadership research to “transition to new perspectives that account for the complex … needs of organizations [occupations, industries]” [38] (p. 2), it is perhaps appropriate to distinguish between theory and practice. As Breunig [39] states, “Theory is often conceived of as an abstract idea or phenomenon. Practice involves an action component that goes beyond the abstraction of theory… experiential and practical knowledge can be employed as a means to understanding” (p. 109). Competency-based leadership models tend to be very praxis-oriented (e.g., [40,41]). However, criticism of competency models has included limitations in the utility of general models across varying contexts [42]. This leads to the question Day et al. [26] pose: “Given the wide variability across competency frameworks, which are the more accurate representations of reality—if any?” (p. 4). It would appear that perhaps the answer to the question resides in the context [29] and the intent [39] associated with leadership models. For example, the Healthcare Leadership Alliance Model proposed by Stefl [43] is specifically intended to be praxis-oriented within a well-defined industry and occupation context. Similar competency-based leadership models exist for nursing [44], higher education [45], and energy production [46]. However, there are no such leadership models that specifically address the competencies associated with effective leadership in the food system.

### 1.5. The Food Systems Leadership Context

The current food system faces many concurrent challenges. At the highest level, food systems must provide nutrition and food security for a growing population while providing livelihoods and doing so in a sustainable manner [23]. The need to develop unique leadership capacities amongst actors within the food system capable of meeting these challenges has never been greater [47,48,49]. As Alexandri et al. [50] state, “the transition toward sustainable food systems requires adequate financial support and training to assist the agri-food sector to comply with the demands of the new era” (p. 25).

As a result, individuals that are engaged in food system-related endeavors must pursue multiple outcomes simultaneously. Specifically, these are the production of calories and commodities, effective advocacy for the industry, and educating the public and policy makers with respect to the impacts and consequences of decisions directed toward agriculture [23,51]. Based on these trends and future mandates, individuals in occupations associated with the food system will need to become more adroit at effectively advocating for and providing education about the agriculture and food industry. To successfully fulfill this mandate, they must act as leaders within their peer groups, organizations, and communities [9,21,52]. “Without leadership and the political will to change the status quo, we won’t be able to overcome the difficulties ahead, and we won’t be able to put these ideas into practice. Success will depend on leaders in industry and government” [53] (para. 10).

The challenges facing the food system and related industries are vast. The individuals involved with the food system must begin to see themselves as leaders and act accordingly, whether through formal or informal channels [21,23,52,54]. However, the ability to address current and future issues using the leadership approaches of the past may prove to be insufficient due to the breadth and depth of the areas of expertise contemporary food system professionals are expected to command [55]. Leaders within the food system must exercise leadership within multiple contexts, from the most proximal interpersonal interactions to the most distal policy-level interactions [9,23,52,55]. 

The boundary spanning of leadership within the context of a nested hierarchical structure represents a context-specific challenge to leaders within the food system [26,29]. Currently, a context-specific [29], practical [39], and competency-based [26] leadership model for food systems leadership is unavailable. 

#### 1.5.1. Leadership at the Interpersonal Level

At the most fundamental level, leadership requires an interaction between at least two individuals [24]. For much of human history, leadership at an interpersonal level was all that was required to exert influence or provide leadership within the agriculture and natural resources area [56]. In more contemporary society, there has been an ongoing need for food system professionals to be able to effectively work on an interpersonal level [54]. Consequently, effective leadership and the ability to navigate a changing environment require the ability to work with others on an interpersonal level (e.g., [40,49]). For example, the ability for food system leaders to act as change agents and establish the appropriate information exchange relationships with their peers is critical to stemming the loss of potentially valuable biodiversity data [57].

#### 1.5.2. Leadership at the Organizational Level

Many food system professionals participate in professional, volunteer, or commodity organizations. For example, the American Farm Bureau Federation (AFBF) “is the country’s largest general farm organization and the leading advocate for farm and ranch families” [58] (para. 1). Participation in organizations with unique and varied mandates, missions, and structures is another change in the scope of food system leader contexts. In the United States alone the Department of Agriculture National Institute of Food and Agriculture [59] recognizes 25 unique National Boards and Councils, 26 Federal Marketing Order Boards and Committees, and 18 State Boards and Committees. The necessity of developing the appropriate skills and abilities to be successful within food system-related organizational environments represents a further shift in the operational realities for those involved in the food system [60]. 

#### 1.5.3. Leadership at the Community Level

Working with the general public and broader communities in which decisions impacting agriculture, natural resources, and food systems are made has been a shift from previous food systems leadership requirements; leading educational initiatives and working within the community context are critical for the agriculture and natural resources industry to remain viable [9,52]: “As urban consumers demand to know more about where their food is coming from, it will be up to farmers and ranchers to educate them” [61] (para. 1). Urbanization represents a major driver in both food system consumption and expectations [62]. Historically, due to the prevalence of agriculture as an occupation, there was a more comprehensive understanding of the consequences of actions directed at the agriculture and natural resource industries [63]. The consequence of a less-informed community making operational decisions for the agriculture and natural resources industry has resulted in unexpected consequences [22]. Providing leadership within their communities has been paramount for those engaged in food systems to ensure the proposed impacts on the industry are better understood [21].

#### 1.5.4. Leadership at the Policy Level

The necessity for food system professionals to operate at a policy level represents an additional shift from the previous expected modes of leadership interaction [21,52]. The trend towards a more direct engagement in policymaking activities from the food systems perspective is a trend which has emerged only within the past few decades [21,64]. The policymaking process is highly complex and involves multiple organizations and agencies: legislatures, administrative agencies, the courts, and even the highest levels of executive policy makers all have vital roles to serve [64]. The leadership capacity to effectively advocate for the needs of food systems across these various entities continues to increase every year [9,23,64]. 

### 1.6. Study Purpose

The purpose of this study was to establish a domain for a food systems leadership model. The study was driven by the following research objectives:

1. To create a comprehensive list of leadership competencies and behaviors associated with effective food systems leadership;
2. To generate a consensus on the specific leadership competencies and behaviors associated with effective food systems leadership;
3. To develop a heuristic thematic grouping of leadership competencies and behaviors associated with effective food systems leadership.

## 2. Materials and Methods

To address the research objectives, a modified Delphi method research design [65] was employed. Specifically, the Delphi method was used to gain experts’ opinions regarding the development of a comprehensive competency framework for agriculture and natural resources leadership development programs. “Delphi has often been used for the purpose of content validation of constructs to be used in quantitative research” [66] (Chapter 8, para. 1). Academics, researchers, and businesses have used the Delphi method extensively for over 50 years [66] to track trends in food supply [67], policy [68], health [69,70], social science [71,72], and science and technology [73]. 

### 2.1. Expert Panel Selection

According to Czinkota and Ronkainen [74], “the selection of the experts is critical to the success of a Delphic study” (p. 152). The Delphi method harnesses the expertise of individuals familiar with the issue of interest [66]; consequently, “individuals comprising the expert panel should represent the research purpose in a way that legitimates the outcome of the Delphi process” [66] (Chapter 6, para. 2). Based on the contextual nature (agriculture and food systems) of the study, expert panelists were identified based on specific selection criteria, as suggested by Okoli and Pawlowski [73]. 

The expertise domain was defined as individuals actively engaged in the leadership development of food system professionals. This group was identified based on recommendations in the leadership literature [26] to undertake leadership studies which are “real-world” (p. 3) in nature and seek to pair scientists with practitioners. Furthermore, the approach addressed the praxis-forward approach recommended by Breunig [39]. To establish the representativeness of practitioner experts, the International Association for Programs of Agricultural Leadership (IAPAL) was selected as an appropriate frame. IAPAL is “a consortium of leadership programs in the USA and several other countries which focus on the leadership development of individuals, communities, and the agricultural and rural industries” [75] (p. 21).

A total of 35 food system-related adult leadership development program directors were invited to serve as experts in the Delphi study, with 32 agreeing to participate. The experts represented programs from Arizona, Arkansas, California, Colorado, Delaware, Florida, Georgia, Illinois, Indiana, Kansas, Kentucky, Louisiana, Maryland, Michigan, Minnesota, Montana, Nebraska, New York, North Dakota, Oklahoma, Pennsylvania, Virginia, Washington, Wisconsin, and Wyoming. Additionally, international program directors from Australia, New Brunswick (Canada), Ontario (Canada), and Scotland also participated. Collectively, the participating directors had provided adult agricultural and food systems leadership development programing for thousands of individuals in the industry [76]. Director experience ranged from less than two years to over 25 years of director experience, helping to ensure a diversity of experiences and perspectives [66]. The panel was composed of 50% female (n = 16) and 50% male (n = 16) participants. 

The expert panel was deemed appropriate based on existing guidance within the literature with respect to the Delphi process. Specifically, the number of panelists, 32, was larger than the median number of panelists, 17, reported in a previous systematic review of Delphi studies [69]. The number of panelists was also consistent with the findings from a more recent Delphi synthesis study [77]. Furthermore, from a panel selection perspective, “The Delphi does not call for expert panels to be representative samples for statistical purposes. Representativeness, it seems, is assessed on the qualities of the expert panel rather than its numbers” [78] (p. 378). Additionally, “experts should be chosen for their work in the appropriate area and credibility with the target audience” (p. 379). Therefore, the expert panel fulfilled the selection criteria associated with the Delphi process. 

Following the third round of the Delphi process, the researcher selected a subset of 11 panelists to further participate in a thematic analysis using an open-sort grouping process [79]. One expert from the Southern region initially sorted the specific component competencies into categories. Ten experts then reviewed and provided input to the proposed thematic coding. The experts were selected to ensure a diversity of geographic representation. 

### 2.2. Instrumentation

The study employed three iterations of the Delphi method. The process and instrumentation were developed based on previous recommendations [80] and empirical studies within the literature [81]. 

The instrument for the first round served as an initial qualitative process to discover the top five behaviors which food system-related (including agricultural and natural resource) leadership development program directors believed program participants should embody upon graduating from a leadership development program [71]. A total of 30 responses were obtained for a 94% response rate. The expert responses were analyzed and aggregated where appropriate [66,71], and a final list of 142 items was generated.

The second round of the Delphi process was designed to capture the expert panel’s level of agreement as a group with the behaviors individually identified in the first round. The level of agreement or disagreement with each item was indicated on a five-point Likert-type scale (1 = strongly disagree, 2 = disagree, 3 = neither agree nor disagree, 4 = agree, and 5 = strongly agree). A mean score ≥ 4.00 was set a posteriori for the item to continue to the third round [66]. A total of 30 responses were obtained for a 94% response rate. A total of 120 behaviors remained after round two.

The third round of the Delphi process was used to establish the expert panel members’ level of consensus with the behaviors retained from the second round. The expert panel was asked to indicate whether they agreed that the behavior should be associated with food systems leadership development programs by marking Yes or No. A consensus level of 80% was set a posteriori. A total of 30 responses were obtained for a 94% response rate. There were 108 items with a consensus of over 80% which were included in the final list of behaviors.

### 2.3. Data Analysis

Data for all three rounds of the Delphi were collected directly via the online survey tool Qualtrics. Once data were entered into the online tool, the results were downloaded, run, and subsequently analyzed using the Statistical Package for the Social Sciences (SPSS), version 21. The data analysis for round one of the Delphi included a grammatical review and update, specifically resolving spelling and grammatical errors [66]. Descriptive statistics were calculated based on the data collected during round two of the Delphi to determine the level of agreement with the behaviors [82]. Finally, descriptive statistics were calculated based on the data collected during round three of the Delphi to determine the consensus amongst panelists across behaviors [82].

At the conclusion of the third round of the Delphi process, a total of 108 behaviors were identified. The retained items were then analyzed thematically by an expert panelist using the constant comparative method [83]. A total of seven primary themes and 35 subthemes were identified. The proposed taxonomy was then sent to a panel of 10 experts as a validation process [84]. Based on expert feedback, updates were made to improve clarity; however, the intents of the specific component competencies remained unchanged. Two pairs of competencies were combined due to content overlap, reducing the total number of specific component competencies to 106.

## 3. Results

During round one of the Delphi process, respondents were asked to identify up to five behavioral outcomes associated with their particular leadership development programs. The respondents identified a total of 142 potential outcomes. Round two of the Delphi process was designed to capture the expert panel’s level of agreement or disagreement that a particular item was an expected behavioral outcome associated with leadership development programs. A total of 120 behaviors remained after round two (Table 1).

The third round of the Delphi process was used to establish the expert panel members’ level of consensus with the behaviors retained following round two. The expert panel was asked to indicate whether they agreed that the behavior should be associated with leadership development programs by marking Yes or No. Following round three, there were 108 items retained (Table 1).

Following round three of the Delphi process, the remaining behaviors were thematically analyzed. Based on the constant comparative method thematic analysis, a structured taxonomy was proposed. Specifically, the analysis included three levels: behaviors (n = 106), subthemes (n = 39), and primary themes (n = 7). Each theme comprised four to nine subthemes. Each subtheme had between one and six behaviors. The final analysis is presented in Table 2.

## 4. Discussion

A three-round Delphi process was employed to identify the expected behavioral outcomes or competencies associated with agriculture and natural resource leadership development programs. A total of 142 competencies were identified during the first round of the Delphi. At the conclusion of the second round of the Delphi process, 120 competencies remained. Following the third and final round of the Delphi process, consensus was achieved for 108 competencies. A final review of the remaining competencies yielded two pairs of redundant items; consequently, 106 competencies remained at the conclusion of the process. 

The 106 competencies were then provided to a group of 11 expert panelists to participate in a thematic open-sort grouping process [79,83]. The resulting taxonomy included three hierarchical levels, including specific component competencies or behaviors (n = 106), broader competency subthemes (n = 39), and primary themes (n = 7). The seven primary leadership themes included action; change; communication; critical thinking, strategic planning, and visioning; interpersonal traits and characteristics; leadership skills; and leadership processes. The results of the Delphi process yielded a number of noteworthy findings relative to the existing leadership literature. 

### 4.1. Contributions to Food Systems Literature

The results of the present study provide a unique contribution to the literature, particularly within the domain of food systems. First, the research was grounded by first establishing the unique characteristics associated with agriculture and food systems as an industry [12,13]. Within this context, the research summarizes relevant leadership literature and establishes the bandwidth–fidelity dilemma [29] within leadership, specifically, the necessity for precision in establishing contexts. Next, the research provides a summary of the fundamental differences between leadership theory and practice, Brueunig [39], and the need for competency-based leadership models to be fundamentally praxis- and utility-forward, particularly within specific contexts [26].

Although the literature dedicated to leadership is vast in both theory and development (see [24,25]), there are no leadership models specifically developed for the agricultural and food system context. However, previous research has found that more theoretical and non-contextual leadership models may fail to meet fit-for-purpose standards [32]. On the contrary, there are numerous such models in other precisely defined industries, such as health care [43], which provide practical and industry-relevant utility. Expanding upon such models, the present research and the proposed Issue Leadership model address this gap.

From a methodological perspective, the approach within the research was novel as it was very praxis-oriented and sought to bridge leadership development practice and leadership model formulation [26]. The Delphi process allowed for the emergence and evaluation of unique behaviors and competencies which were then thematically grouped. The process was similar to the taxonomic process of “lumping and splitting” [85] wherein descriptive and inclusive but non-overlapping categories are proposed to order data. The constant comparative analysis [83] and validation [84] provided a heuristic model to represent the study results.

### 4.2. Contributions to Food Systems Practice

From a practical perspective, the research provides a practical, competency-based leadership model for use within food systems. For example, the action factor is unique and not specifically established within comprehensive leadership models [27,28]. Similarly, the concept of action is not present in models for transformational [33], servant [34], or authentic [36] leadership. This finding is noteworthy as action fundamentally underlies the need for leadership. A recommendation for practice would be to design leadership development opportunities for individuals engaged in agricultural- or food system-related roles to take on leadership responsibilities. Additionally, the prominence of advocacy and political and civic engagement are consistent with the action-related competencies one may require within the agricultural and food system industry [21].

Unlike action, change was identified as a primary leadership area within Yukl et al.’s [28] comprehensive model. This finding indicates the centrality of the role of leaders in the change process. However, change was not specifically identified in the factors associated with transformational [33], servant [34], or authentic [36] leadership. The findings from the research indicate effective agricultural and food system leaders must be willing to embrace change and serve as catalysts for change [86]. A recommendation is for agricultural and food system leaders to receive specific training on concepts such as innovation [41] and to become more comfortable and confident in their role in supporting change. This competency area will be of fundamental importance to ensure an adaptable industry [9,23].

The number of competencies and behaviors associated with the communication factor of the proposed domain was unanticipated relative to the scarceness of specific competencies within the leadership literature. Specifically, the types and modalities of communication competence are notably absent, particularly within a food system context. Written and oral communication, listening, persuasive speaking, and media relations all emerged as important competencies within food systems. Although the ability to inspire followers has long been associated with transformational leadership [87], without an effective means to do, so the potential remains limited. Therefore, a developed capacity to convey one’s intention through the ability to effectively communicate is paramount [25]. A recommendation would be to provide applied communication competency development training for agricultural and food system leaders. For example, developing the ability to make sound and reasonable arguments and speak persuasively is critical when leaders interact with peers, policy makers, or community members [21,63].

Critical thinking, strategic planning, and visioning included numerous competencies and behaviors which had equivalents within the leadership literature. For example, problem solving [88], decision making [25] and goal setting [89] are established in many existing leadership models. However, global and systems thinking and critical thinking tend to be absent. The results indicate that without the capacity to extend beyond causal thinking and problem solving, a leader’s effectiveness within the food system may be limited [9,49]. A recommendation would be to focus on developing systems thinking as this is likely a competency absent from other leadership models. As parts of a complex and integrated system, effective agriculture and food system leaders will need to understand a range of both direct and indirect effects [23].

Interpersonal traits and characteristics included many competencies and behaviors found in existing leadership models. For example, confidence [90], empathy [34], ethics [91], integrity [92], and self-awareness [88] are frequently identified in the leadership literature; however, rarely are all competencies proposed within a single framework. The more inclusive set of competencies and behaviors included in the proposed model extends upon a more limited set of factors in many models, a frequent source of model criticism [26]. A recommendation would be to provide agricultural and food system leaders with access to a variety of developmental opportunities across the range of emergent themes. Specifically, defining a development roadmap for leaders may help to ensure that individuals understand the breadth of competencies and do not focus only on developing a limited number of competency areas.

An analysis of the leadership skill theme and the subthemes of the proposed domain provides an example of the challenge associated with proposing a leadership model for the food system. Specifically, there are numerous leadership competencies which are necessary to provide effective leadership across interpersonal, organizational, community, and policy levels which do not necessarily lend themselves to informative thematic names. The leadership skill was intended to encompass leadership behaviors and competencies which tend to originate within the leader themselves. For example, recognizing values may be associated with motivating others from an interpersonal perspective; however, recognizing the values of others may also be beneficial for setting policy agendas. Regardless of the intended context, it is the leader’s responsibility to actively seek to understand and recognize the values of others. The core of the theme is to acknowledge the agency of the leader in the application of the behavior or competency. Effective food system leaders should engage in leadership skill-related activities to develop both an awareness of and a desire to cultivate such capacities. Although there were limited equivalents across other analyzed leadership models [33,34,36], a recommendation would be for agriculture and food system leaders to work on developing leadership skill competencies across a range of environments. For example, conflict management may require unique approaches when engaging interpersonally, organizationally, within a community, or politically. Finding opportunities to engage in multiple environments will help provide a depth of expertise. 

Finally, the leadership process theme shared similar characteristics with the leadership skill theme, particularly when analyzing the challenge in classifying and thematically grouping behaviors and competencies. However, unlike the leadership skill theme, in which behaviors and subthemes were more leader-centric and standalone, the leadership process theme was more dynamic in nature. Specifically, the subthemes and behaviors require an interaction between the leader and follower. For example, trust building is a social phenomenon which only occurs between individuals. Furthermore, the ability to build trust with others undergirds many of the contexts (interpersonal, organizational, community, and policy) in which food system leaders engage. Additionally, the concept of servant leadership emerged, which is equivalent to the model proposed by Greenleaf [34]. A recommendation would be for agriculture and food systems leaders to focus on the service aspects of their role and how leadership processes can improve effectiveness when working with others.

### 4.3. Limitations

The intent of the current study was to establish a domain for a food systems leadership model; however, it is important to identify and address a number of limitations associated with the process to aid in result interpretation. First, any form of Delphi research is dependent upon the expertise of the panelists contributing to the process [66]: “if panelists are misinformed about a topic, the use of Delphi may only add confidence to their ignorance” [93] (p. 39). To mitigate this issue, panelists were purposively selected based on their expertise and experience in adult food system-related leadership development programs. However, despite the selection and inclusion criteria, the results were limited to the panel responses. Therefore, there is the potential for behaviors and subsequent themes and subthemes to be impacted accordingly. Additionally, the majority of the expert panelists represented agriculture and food systems leadership development programs located in the United States. A recommendation would be to examine the applicability of the results outside of the United States. 

A second primary limitation is associated with the manner in which the Delphi output, the 106 unique behaviors or competencies, were thematically grouped and analyzed. From this perspective, it is important to note the intent of the thematic analysis process was heuristic in nature. As a domain-related research process, the intent was to provide an interpretative lens to facilitate understanding. The use of experts and of a member checking the results was completed as recommended in the literature [84]; however, interpretations of the thematic analysis should be conducted within this context. An associated recommendation would be to apply the findings from the current study to inform future food systems leadership research. 

Lastly, the results and the discussion associated with the present study were limited to descriptive statistics and a proposed model based on the thematic analysis. The intent of the present study was to establish a domain for a food systems leadership model. Therefore, the analyses associated with the study were very basic in nature. A recommendation for future research and practice would be to use the results of the current study as the foundation for more sophisticated analyses. For example, developing and validating an Issue Leadership scale may further extend the utility of the present findings. 

## 5. Conclusions

Although numerous theories of leadership are available within the literature, there has been little focus on effective leadership within the food system [25]. The purpose of the research was to create a comprehensive list of leadership competencies and behaviors associated with effective food systems leadership, generate a consensus on the specific leadership competencies and behaviors associated with effective food systems leadership, and develop a heuristic thematic grouping of leadership competencies and behaviors associated with effective food systems leadership. The results of the present study address this gap in the literature and research objectives. Specifically, 106 specific competencies or behaviors for effective agricultural and food systems leadership were identified. These items achieved consensus amongst a panel of food systems leadership development experts. The heuristic thematic grouping of the items resulted in a food systems leadership model named Issue Leadership which is composed of seven primary themes and 39 subthemes. This is the first food systems leadership model proposed in the literature. The approach addresses one of the main criticisms leveled at leadership development: that leadership is presumed to be universal agnostic of situation [26,94]. The results of the present study provide a context-specific leadership model upon which to further examine leadership within the food system. 

From an international perspective, the current study provides important considerations for policy. Working from the United Nations Sustainable Development Goals (UN SDGs) [95] as a framework, the role of leadership in addressing agriculture-related SDGs is evident. For example, Goal 2: Zero Hunger, is directly related to the food system. The UN has developed recommendations for businesses to align with the SDGs [96], which specifically state, “A key way to address hunger is by improving productivity and sustainability, market access, and access to opportunities for upgrading into more value-added activities” (p. 18). Similarly, the UN [97] has identified key characteristics associated with CEO and board members who have demonstrated records of integrating sustainability into business strategy. The study found that the leaders have common leadership attributes including multilevel system thinking, stakeholder inclusion, disruptive innovation, and long-term activation. The current study provides an actionable framework to empower all levels of the food system value chain, including the producers directly engaged in production agriculture. Adopting a common framework for individual development and empowerment may aid in improving the ability to improve the productivity and sustainability of the food system. Future research applying the Issue Leadership framework within the food system is recommended to explore the dimensionality and applicability of the framework within a variety of contexts.

## Figures and Tables

**Table 1:** Delphi round two results (1 = strongly disagree; 5 = strongly agree) (*n* = 32) and Delphi round three level of consensus (*n* = 30).

Item	1	2	3	4	5	Mean	Consensus (%)
Act with integrity	0	0	0	5	25	4.83	100
Demonstrate proficiency in communication skills through effective interpersonal interaction	0	0	0	10	20	4.67	100
Able to appreciate different perspectives and opinions as well as an openness to differences	0	0	1	8	21	4.67	100
Confident to step forward into a leadership role at the organization, local, state/province/territory, national or international level	0	0	1	8	21	4.67	100
Demonstrate critical thinking by asking appropriate questions and applying reflective thinking skills to find solutions	0	0	1	10	19	4.60	100
Confident in self and abilities	0	0	1	10	19	4.60	100
Foster collaboration by promoting cooperative goals and building trust	0	0	1	10	19	4.60	100
Demonstrate problem-solving skills	0	0	0	15	15	4.50	100
Act as an agent of change	0	0	2	11	17	4.50	100
Willing to get involved and engaged in organizational and community endeavors	0	0	2	12	16	4.47	100
Demonstrate a willingness to take risks and try new ideas and approaches	0	0	1	15	14	4.43	100
Able to work as part of a team	0	0	0	6	24	4.80	96.67
Adaptable and collaborative and can work with diversity	0	0	1	6	23	4.73	96.67
Be open to new ideas	0	0	0	9	21	4.70	96.67
Challenge the process—search for opportunities by seeking innovative ways to change, grow, and improve	0	0	1	7	22	4.70	96.67
Respect others opinions	0	0	1	8	21	4.67	96.67
Lead by example	0	0	2	7	21	4.63	96.67
Self-aware of leadership behaviors, strengths, and areas for improvement	1	0	0	8	21	4.60	96.67
Know personal strengths and weaknesses	0	0	1	12	17	4.53	96.67
Self-aware of individual skills: life, interpersonal, and business	1	0	0	11	18	4.50	96.67
Take initiative to proactively solve problems	0	0	1	14	15	4.47	96.67
Demonstrate the interpersonal skills necessary to build a personal and professional network of peers that can be leveraged in a variety of situations	1	0	0	13	16	4.43	96.67
Give of their time and talents	0	0	2	14	14	4.40	96.67
Evaluate self-performance	0	0	3	13	14	4.37	96.67
Demonstrate critical thinking by challenging existing ways of thinking	0	1	3	13	13	4.27	96.67
Demonstrate critical thinking by integrating multiple perspectives and systems	0	2	2	12	14	4.27	96.67
Demonstrate an awareness of public issues through, reading, research, questioning and writing	1	0	4	11	14	4.23	96.67
Build better teams based on team members’ strengths	0	1	2	17	10	4.20	96.67
Be attentive to group process, by being aware of the stages of group growth and drawing out feedback from all group members	1	1	6	9	13	4.07	96.67
Demonstrate stewardship	1	1	3	17	8	4.00	96.67
Demonstrate an ability to think globally and act locally	0	0	4	11	14	4.34	96.55
Take accountability for their actions	0	0	0	9	21	4.70	93.33
Expand networks and connections to have greater influence as local leaders	0	0	0	11	19	4.63	93.33
Demonstrate lifelong learning through continued personal and professional development	0	0	1	10	19	4.60	93.33
Make decisions in an ethical and values based manner by being inclusive, constructive, and enabling	0	0	1	13	16	4.50	93.33
Expand networks and connections to have greater influence as state/province/territory leaders	0	0	1	13	16	4.50	93.33
Demonstrate competence by expanding and improving knowledge and skills needed to be a responsive leader	0	1	0	13	16	4.47	93.33
Foster deep networks within stakeholder groups both inside and outside of the agricultural industry	0	0	4	9	17	4.43	93.33
Recognize contributions of others by showing appreciation for individual excellence	0	0	2	13	15	4.43	93.33
Demonstrate clear personal values, set an example by aligning actions with shared values	1	0	1	12	16	4.40	93.33
Influence policy makers	0	0	1	16	13	4.40	93.33
Identify state/province/territory issues and recognize the social, cultural, economic, political, and environmental connections	0	0	1	16	13	4.40	93.33
Stay informed on state/province/territory agricultural issues	0	0	1	17	12	4.37	93.33
Stay informed on the political process	0	0	1	17	12	4.37	93.33
Identify innovative solutions to challenges	0	0	2	15	13	4.37	93.33
Identify local issues and recognize the social, cultural, economic, political, and environmental connections	0	0	3	14	13	4.33	93.33
Confident working with the media	0	1	0	19	10	4.27	93.33
Apply unique talents purposefully	0	2	1	15	12	4.23	93.33
Participate in local leadership positions	0	1	3	14	12	4.23	93.33
Able to analyze a public or organizational issue and determine a plan of action	0	0	2	19	9	4.23	93.33
Understand the legislative process at the national level	0	1	1	18	10	4.23	93.33
Able to lead groups to a common end	0	0	4	16	10	4.20	93.33
Demonstrate inspired motivation to lead and persuade others	1	0	0	20	9	4.20	93.33
Demonstrate public issues knowledge	0	0	3	19	8	4.17	93.33
Understand the legislative process at the state/province/territory level	0	1	1	20	8	4.17	93.33
Lead diverse groups, organizations, and communities in developing solutions to address local challenges	0	0	4	18	8	4.13	93.33
Demonstrate advocacy skills	0	2	3	15	10	4.10	93.33
Mentor others to improve their skills and apply them to leadership roles	0	1	5	17	7	4.00	93.33
Demonstrate proficiency in communication skills through effective public speaking	0	1	1	14	14	4.37	93.10
Demonstrate an ability to be adaptable and collaborative by negotiating and resolving conflict	1	0	1	14	14	4.33	93.10
Demonstrate a broadened perspective on major issues affecting agriculture	0	0	4	15	11	4.23	93.10
Stay informed on local agricultural issues	0	0	3	13	14	4.37	92.86
Develop, refine, and use networks appropriately and effectively	0	0	0	8	22	4.73	90.00
Enable others to act—strengthen others by sharing power and discretion	0	0	0	14	16	4.53	90.00
Demonstrate an ability to be adaptable and collaborative by working with diversity	0	0	3	11	16	4.43	90.00
Act reflectively and openly to learning while seeking wisdom	0	1	3	9	17	4.40	90.00
Create and communicate a vision for the future of an organization or community	0	0	3	12	15	4.40	90.00
Expand understanding of global economic, political, cultural, and social systems and how they affect agriculture	0	0	3	12	15	4.40	90.00
Become more involved in the community	0	0	3	12	15	4.40	90.00
Create and communicate a vision for the future of an organization or community	0	0	3	12	15	4.30	90.00
Identify national issues and recognize the social, cultural, economic, political, and environmental connections	0	0	3	13	14	4.37	90.00
Able to make sound and reasonable verbal arguments	0	1	0	16	13	4.37	90.00
Demonstrate an attitude of servant leadership	1	0	3	9	17	4.37	90.00
Influence local public policy	0	0	1	17	12	4.37	90.00
Facilitate discussions between individuals	0	0	3	14	13	4.33	90.00
High level of self-efficacy, able to produce desired results and make change happen	1	0	2	14	13	4.27	90.00
Work within local, state/province/territory and national government structures to shape opinions and advocate policy	0	1	3	13	12	4.24	90.00
Demonstrate an ability to tap all resources available to tackle an issue	0	0	4	15	11	4.23	90.00
Understand the legislative process at the local level	0	1	2	18	8	4.14	90.00
Be an active participant in community activities	0	1	7	13	9	4.00	90.00
Deep listening skills	0	0	2	9	19	4.57	86.67
Demonstrate a respect for conflict as a means to learn, grow, and change	0	0	2	11	17	4.50	86.67
Demonstrate initiative through the ability to make things happen	0	0	0	17	13	4.43	86.67
Able to deal with conflict as it relates to various opinions around agriculture and environmental issues	0	0	0	18	12	4.40	86.67
Effective advocate for agriculture	0	0	4	10	16	4.40	86.67
Expand understanding of national economic, political, cultural, and social systems and how they affect agriculture	0	0	4	11	15	4.37	86.67
Develop networks with program alumni who care about the agricultural industry	0	0	4	13	13	4.30	86.67
Engage in collaborative decision making and creative problem solving with agriculture as a context for broader issues	0	1	2	14	13	4.30	86.67
Stay informed on national agricultural issues	0	0	3	16	11	4.27	86.67
Participate in community groups	0	0	4	15	11	4.23	86.67
Able to lead individuals to a common end	0	0	2	19	9	4.23	86.67
Act inclusively with others	1	1	2	12	14	4.23	86.67
Participate in interest (commodity, association) groups	0	1	3	14	12	4.23	86.67
Stay informed on global agricultural issues	0	0	3	18	9	4.20	86.67
Demonstrate the ability to be persuasive	0	1	1	19	9	4.20	86.67
Influence state/province/territory public policy	0	0	1	22	7	4.20	86.67
Identify international issues and recognize the social, cultural, economic, political, and environmental connections	0	0	3	18	9	4.20	86.67
Act empathetically	0	1	6	13	10	4.07	86.21
Demonstrate the skills necessary to act as a leader within the agricultural industry	1	0	4	11	14	4.23	83.33
Set goals consistently and make them measurable	0	0	4	15	11	4.23	83.33
Act as a resource for the local agricultural industry	0	1	4	15	10	4.13	83.33
Serve as recognizable ambassador for agriculture	0	0	7	12	11	4.13	83.33
Active civic engagement	1	0	4	14	11	4.13	83.33
Demonstrate an awareness of agriculture on an international level	0	0	6	15	9	4.10	83.33
Understand the political scene and political decision making process at all levels	0	2	2	17	9	4.10	83.33
Able to make sound and reasonable written arguments	0	2	3	15	10	4.10	83.33
Demonstrate persistence in overcoming defeats	0	2	2	18	8	4.07	83.33
Lead diverse groups, organizations, and communities in developing solutions to address state/province/territory challenges	0	0	5	18	7	4.07	83.33
Be involved in the policy process at the local level	0	1	3	17	9	4.13	80.00
Act as a resource for the state/province/territory agricultural industry	0	1	4	15	10	4.13	80.00
Comfortable educating media, urban audiences, decision makers, and others on issues affecting agricultural issues	0	2	3	14	11	4.13	80.00
Participate in state/province/territory leadership positions	0	1	4	18	7	4.03	80.00
Demonstrate critical thinking by tolerating paradox and ambiguity	0	1	6	15	8	4.00	79.31
Navigate shifting social, environmental, business, political and structural drivers shaping the agricultural industry	1	0	4	16	9	4.07	76.67
Demonstrate passion and excitement for the agricultural industry	1	0	7	11	11	4.03	75.86
Influence national public policy	0	0	3	20	6	4.10	73.33
Place service to others above self	0	1	5	14	10	4.10	73.33
Have a voice in agriculture and educate about agricultural industry	0	2	5	13	10	4.03	73.33
Demonstrate enthusiasm about the agricultural industry	0	1	7	13	9	4.00	73.33
Be involved in the policy process at the state/province/territory level	0	1	4	18	7	4.03	70.00
Are ambitious in their actions	0	2	6	11	11	4.03	63.33

**Table 2:** Constant Comparative Method Thematic Analysis Results (*n* = 106).

Themes	Subthemes	Behaviors
Action	Advocacy	Effective advocate for agriculture; Work within local, state/province/territory and national government structures to shape opinions and advocate policy; Work within local, state/province/territory and national government structures to shape opinions and advocate policy related to agriculture and the environment
	Assume Leadership Roles and Increase Involvement	Act as a resource for the local agricultural industry; Give of their time and talents; Participate in community groups; Participate in interest (commodity, association) groups
	Mentorship	Mentor others to improve their skills and apply them to leadership roles
	Networking	Develop networks with program alumni who care about the agricultural industry; Develop, refine, and use networks appropriately and effectively; Expand networks and connections to have greater influence as local leaders; Expand networks and connections to have greater influence as state/province/territory leaders; Foster deep networks within stakeholder groups both inside and outside of the agricultural industry
	Political And Civic Engagement	Active civic engagement; Expand understanding of global economic, political, cultural, and social systems and how they affect agriculture; Expand understanding of national economic, political, cultural, and social systems and how they affect agriculture
	Serve As a Resource	Apply unique talents purposefully; Assess financial and market viability of an organization
Change	Change Agent	Act as an agent of change; Create new approaches/ideas
	Innovation	Be open to new ideas; Challenge the process—seek innovative ways to change, grow, and improve; Identify innovative solutions to challenges
	Risk-Taking	Demonstrate a willingness to take risks and try new ideas and approaches; View challenges as something to be mastered rather than avoided
	Self-Efficacy	Believe in my ability to succeed or perform well; High level of self-efficacy, able to produce desired results and make change happen
Communication	Listening	Active listening skills; Facilitate discussions between individuals
	Media Relations	Confident working with the media
	Persuasive Speaking	Demonstrate proficiency in communication skills through effective public speaking; Demonstrate the ability to be persuasive
	Written And Oral	Able to make sound and reasonable verbal arguments; Able to make sound and reasonable written arguments; Comfortable using various communication technologies, including social media
Critical Thinking, Strategic Planning and Visioning	Critical Thinking	Demonstrate an ability to seek out multiple views and take a strategic overview; Demonstrate critical thinking by asking appropriate questions and applying reflective thinking skills to find solutions; Demonstrate critical thinking by challenging existing ways of thinking; Demonstrate critical thinking by integrating multiple perspectives and systems
	Decision Making	Able to analyze a public or organizational issue and determine a plan of action; Demonstrate an ability to act decisively
	Global And Systems Thinking	Demonstrate an ability to think globally and act locally; Demonstrate an awareness of agriculture on an international level; Identify international issues and recognize the social, cultural, economic, political, and environmental connections; Identify local issues and recognize the social, cultural, economic, political, and environmental connections; Identify national issues and recognize the social, cultural, economic, political, and environmental connections; Identify state/province/territory issues and recognize the social, cultural, economic, political, and environmental connections
	Goal Setting (Or Visioning)	Communicate a vision for the future of an organization or community; Facilitate creation of a vision for the future of an organization or community; Set goals consistently and make them measurable
	Problem Solving	Demonstrate problem-solving skills; Engage in collaborative decision making and creative problem solving
	Stakeholder Assessment	Assess the impact of your actions on the various stakeholders involved
Interpersonal Trait and Characteristic	Confidence	Confident in self and abilities; Confident to step forward into a leadership role at the organization, local, state/province/territory, national or international level
	Empathetic	Act empathetically; Respect others opinions
	Ethical	Make decisions in an ethical and values based manner by being inclusive, constructive, and enabling
	Initiative	Demonstrate initiative through the ability to make things happen; Take initiative to proactively solve problems
	Integrity	Act with integrity; Lead by example; Take accountability for actions of self and organization
	Life-Long Learning	Demonstrate a broadened perspective on major issues affecting agriculture; Demonstrate competence by expanding and improving knowledge and skills needed to be a responsive leader; Demonstrate life-long learning through continued personal and professional development
	Motivation	Demonstrate inspired motivation to lead and persuade others
	Self- Awareness	Demonstrate the interpersonal skills necessary to build a personal and professional network of peers that can be leveraged in a variety of situations; Evaluate self-performance; Know personal strengths and weaknesses; Seek wisdom through reflection and openness to learning; Self-aware of individual skills: life, interpersonal, and business; Self-aware of leadership behaviors, strengths, and areas for improvement
	Understanding And Appreciating Diversity	Able to appreciate different perspectives and opinions as well as an openness to differences; Act inclusively with others; Adaptable and collaborative and can work with diversity; Demonstrate an ability to be adaptable and collaborative by working with diversity
Leadership Skill	Conflict Management	Able to deal with conflict as it relates to various opinions around agriculture and environmental issues; Demonstrate a respect for conflict as a means to learn, grow, and change; Demonstrate an ability to be adaptable and collaborative by negotiating and resolving conflict
	Fostering And Enabling Others	Delegate responsibilities when appropriate; Demonstrate the skills necessary to act as a leader within the agricultural industry; Enable others to act—strengthen others by sharing power and discretion; Lead diverse groups, organizations, and communities in developing solutions to address local challenges; Lead diverse groups, organizations, and communities in developing solutions to address state/province/territory challenges
	Issue Awareness	Demonstrate an awareness of public issues through, reading, research, questioning and writing; Stay informed on global, national, state/province/territory, local agricultural issues
	Political Process Awareness	Assess the political scene and political decision making process at all levels; Understand the legislative process at the local, state/province/territory, national level
	Recognizing Values	Demonstrate clear personal values, set an example by aligning actions with common values of organization; Demonstrate stewardship
Leadership Process	Collaboration	Able to lead individuals and groups to a common end; Demonstrate an ability to tap all resources available to tackle an issue
	Group/Team Dynamics	Be attentive to group process, by being aware of the stages of group growth and drawing out feedback from all group members; Build better teams based on team members’ strengths; Recognize contributions of others by showing appreciation for individual excellence
	Influence	Influence local, state/province/territory public policy; Influence policy makers
	Servant Leadership	Demonstrate an attitude of servant leadership
	Trust Building	Foster collaboration by building trust and promoting cooperative goals

## Data Availability

The data are not publicly available due to confidentiality restrictions. Please reach out to the corresponding author for questions related to data availability.
